# Targeted Next-Generation Sequencing Identifies Separate Causes of Hearing Loss in One Deaf Family and Variable Clinical Manifestations for the p.R161C Mutation in *SOX10*

**DOI:** 10.1155/2020/8860837

**Published:** 2020-08-28

**Authors:** Xiaoyu Yu, Yun Lin, Hao Wu

**Affiliations:** ^1^Department of Otorhinolaryngology-Head and Neck Surgery, Shanghai Ninth People's Hospital, Shanghai Jiao Tong University School of Medicine, Shanghai, China; ^2^Ear Institute, Shanghai Jiao Tong University School of Medicine, Shanghai, China; ^3^Shanghai Key Laboratory of Translational Medicine on Ear and Nose Diseases, Shanghai, China

## Abstract

Hearing loss is the most common sensory deficit in humans. Identifying the genetic cause and genotype-phenotype correlation of hearing loss is sometimes challenging due to extensive clinical and genetic heterogeneity. In this study, we applied targeted next-generation sequencing (NGS) to resolve the genetic etiology of hearing loss in a Chinese Han family with multiple affected family members. Targeted sequencing of 415 deafness-related genes identified the heterozygous c.481C>T (p.R161C) mutation in *SOX10* and the homozygous c.235delC (p.L79Cfs∗3) mutation in *GJB2* as separate pathogenic mutations in distinct affected family members. The *SOX10* c.481C>T (p.R161C) mutation has been previously reported in a Caucasian patient with Kallmann syndrome that features congenital hypogonadotropic hypogonadism with anosmia. In contrast, family members carrying the same p.R161C mutation in this study had variable Waardenburg syndrome-associated phenotypes (hearing loss and/or hair hypopigmentation) without olfactory or reproductive anomalies. Our results highlight the importance of applying comprehensive diagnostic approaches such as NGS in molecular diagnosis of hearing loss and show that the p.R161C mutation in *SOX10* may be associated with a wide range of variable clinical manifestations.

## 1. Introduction

Hearing loss is the most prevalent neurosensory impairment in humans, affecting over half a billion people worldwide [1, 2]. In a mammals' inner ear, cochlear hair cells (HCs) take responsibility to convert the mechanical sound waves into electrical signals [3–5], which make the HCs very important for hearing function. Many previous reports have already shown that HCs can be injured due to genetic factors, noise, ototoxic drugs, aging, or inflammation [6–13]; and it is estimated that 50%-60% of early-onset hearing loss is due to genetic factors [14, 15]. Based on the association with other clinical features, approximately 70% of genetic hearing loss is nonsyndromic and 30% is syndromic. Currently, more than 100 genes for nonsyndromic hearing loss have been identified, and over 700 different forms of syndromic hearing impairment have been described [16, 17]. The extremely high genetic and phenotypic heterogeneity sometimes makes the diagnosis of genetic hearing loss challenging.

Mutations in *SOX10* have been associated with various forms of syndromic hearing loss. SOX10 is a transcription factor involved in cell fate determination and cell lineage development, especially in the forming and differentiation of the neural crest [18]. A variety of mutations in *SOX10* may result in various developmental defects including type II (WS2, OMIM 611584) and type IV (WS4, OMIM 613266) Waardenburg syndrome (WS) featuring auditory and pigmentary abnormalities, with the latter also exhibiting short-segment Hirschsprung disease (HD, OMIM 142623) [19–22]. Recently, mutations in *SOX10* have been identified in a few patients with Kallmann syndrome (KS, OMIM 308700) with deafness, which is characterized by hypogonadotropic hypogonadism, anosmia and hearing loss [23–26]. However, it remains unknown if there is a specific genotype-phenotype correlation between certain *SOX10* mutations and Kallmann syndrome.

In the present study, we applied targeted NGS to identify the genetic etiology of hearing loss in a moderate-sized Chinese Han family with apparently complex inheritance. In different affected family members, we identified separate genetic causes in recessive mutation in the *GJB2* gene and dominant mutation in the *SOX10* gene. Despite the fact that *SOX10* c.481C>T (p.R161C) mutation was previously associated with Kallmann syndrome, family members with this mutation in our study had either normal or only WS2 (hearing loss and hair hypopigmentation) phenotype, indicating a rather variable clinical manifestation.

## 2. Subjects and Methods

### 2.1. Subjects and Clinical Assessments

**Figure 1:**
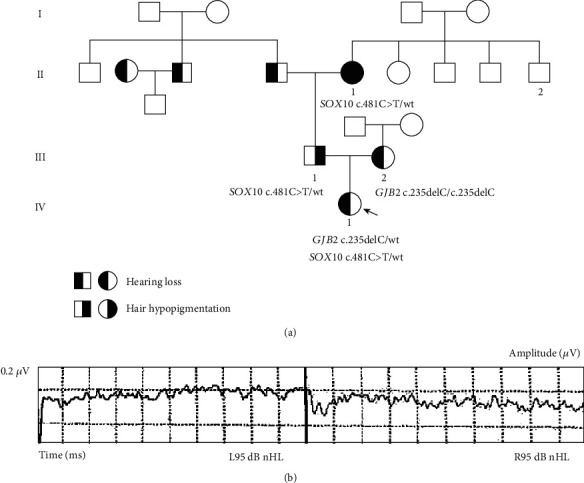
Pedigree of the family and clinical findings of the proband IV-1: (a) pedigree and genotype showing the c.481C>T (p.R161C) mutation in *SOX10* and the c.235delC (p.L79Cfs∗3) mutation in *GJB2*; (b) auditory evoked potentials showing bilateral profound sensorineural hearing loss.

The proband (Figure 1(a), IV-1) with bilateral profound sensorineural hearing loss was enrolled through the Department of Otorhinolaryngology at Shanghai Ninth People's Hospital. Four other subjects (II1-1, II1-2, II-1, and II-2) from the four-generation family and 100 Chinese Han normal hearing controls were also included in this study. The clinical evaluation included a detailed medical history questionnaire and a thorough physical examination. Auditory evaluations were performed in all participants, including otoscopic examination, otoacoustic emissions (OAEs), auditory evoked potentials (AEPs), or pure-tone audiometry. High-resolution computerized tomography (CT) scan of the temporal bone was performed in proband IV-1. Sense of smell was evaluated by self-report, questioning, or olfactory tests. Blood sample was collected from the proband and her family members, and total DNA was extracted from peripheral blood leukocytes using standard protocols.

### 2.2. Ethics Statement

Written informed consent was obtained from all study participants or their guardians. All experimental procedures in this study were approved by the Ethics Committee of Shanghai Ninth People's Hospital, Shanghai Jiao Tong University School of Medicine.

### 2.3. Targeted Genomic Enrichment and Massively Parallel Sequencing

The quality and quantity of genomic DNA were assessed by gel electrophoresis and spectrophotometry. Libraries were prepared using the Illumina standard protocol. Targeted enrichment of all exons and flanking splicing sites of 415 genes implicated in sensorineural hearing loss (for the list of genes, see Supplementary Table S1) was completed using MyGenostics Gencap™ capture kit (MyGenostics, Baltimore, MD, USA) following the manufacturer's protocol. The enrichment libraries were sequenced on Illumina NextSeq 500 sequencer.

### 2.4. Bioinformatics Analysis

The high-quality reads were mapped to the human genome sequence (hg19) with a Burrows-Wheeler-Alignment Tool, and GATK HaplotypeCaller was used to detect small insertions or deletions (InDels) and Single Nucleotide Variants (SNVs) [27, 28]. The identified SNVs and InDels were then annotated using the ANNOVAR software [29]. The missense, frameshift, nonsense, and splicing variants with a minor allele frequency < 1% were further interrogated as candidate pathogenic mutations. For allele frequencies, we used 1000 Genomes (http://www.1000genomes.org/), ESP6500 (http://evs.gs.washington.edu/EVS/), and ExAC (http://exac.broadinstitute.org/) databases. An ensemble tool REVEL (rare exome variant ensemble learner) was used to predict the pathogenicity of missense variants [30]. The ClinVar database and Human Gene Mutation Database (HGMD) were used to further annotate known pathogenic variants. Pathogenicity of the candidate variants was interpreted following American College of Medical Genetics and Genomics (ACMG) standards and guidelines 2015 [31].

### 2.5. Sanger Sequencing

The candidate variants in *SOX10* and *GJB2* gene were amplified by polymerase chain reaction (PCR) and analyzed by Sanger sequencing. The primer sequences for PCR amplification are provided in Supplementary Table S3.

## 3. Results

### 3.1. Clinical Findings

The female proband VI-1 was born from nonconsanguineous parents (Figure 1(a)). She failed the neonatal hearing screening, and further examination revealed bilateral profound sensorineural hearing loss (>95 dB, Figure 1(b)). Her computed tomography scan of the temporal bone revealed slight dilation of the posterior semicircular canals in both ears. When examined at 7 years of age, she had normal dark irides, normal fundus oculi, and no pigmentary alterations in the skin or hair. Dystopia canthorum, limb anomaly, and Hirschsprung disease were absent. Neurological examination was normal.

The proband's mother III-2 had prelingual, bilateral, profound hearing loss. Interview and visual inspection of III-2 did not find pigmentation defects and musculoskeletal anomalies. The father III-1 had normal hearing but reported to develop a white forelock at approximately 8 years of age and prematurely gray hair at 20 years of age. The paternal grandmother II-1 had severe congenital bilateral hearing loss and reported a frontal white forelock and premature graying of hair since approximately 15 years of age. All family members reported normal sense of smell, which was confirmed by olfactory tests. Both III-2 and II-1 had normal puberty and spontaneous pregnancy.

### 3.2. Genetic Analysis Results

**Figure 2:**
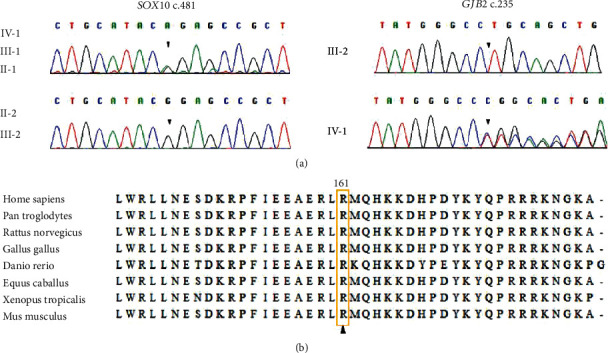
The *GJB2* c.235delC (p.L79Cfs∗3) and the *SOX10* c.481C>T (p.R161C) mutations identified in the family. (a) Sequence chromatogram showing the genotyping results of the family members. The proband IV-1, her father III-1, and the grandmother II-1 had heterogeneous *SOX10* c.481C>T (p.R161C, arrow) mutation. The mother III-2 carried a homozygous *GJB2* c.235delC (p.L79Cfs∗3) mutation. (b) Alignment of SOX10 sequences from various species showing conservation of the arginine residue at position 161.

Targeted next-generation sequencing of 415 deafness-related genes identified a homozygous c.235delC (p.L79Cfs∗3) variant in *GJB2* as the pathogenic cause of hearing loss for the mother III-2 (Figure 2(a)). In the proband IV-1 and her father III-1, we detected 10 and 12 rare (MAF < 0.01 in public databases) heterogeneous nonsynonymous variants, respectively (Supplementary Table S2). Of these, the c.481C>T (p.R161C) variant in *SOX10* has been previously reported resulting in Kallmann syndrome in a Caucasian patient [32]. It substitutes a well-conserved arginine by cystine in the high-mobility group (HMG) domain of SOX10 (Figure 2(b)). This variant was not observed in genotyping of 100 Chinese Han normal controls and was predicted as damaging by in silico assessment with REVEL [30]. According to the 2015 ACMG guideline, c.481C>T (p.R161C) in *SOX10* was classified as likely pathogenic (PS1+PM2). Sanger sequencing validated the presence of this variant in IV-1, III-1, and II-1, three individuals with WS-associated phenotypes (Figure 2(a)).

## 4. Discussion

The cause of hearing loss is extremely heterogeneous, and in many regions of the world, deaf people tend to marry with each other to form rather complex deaf families [33–40]. In one such family, we identified two separate genetic causes of hearing loss in distinct affected members, including the recessive c.235delC (p.L79Cfs∗3) mutation in *GJB2* (III-2) and the dominant c.481C>T (p.R161C) mutation in *SOX10* (II-1, III-1, and IV-1). While the c.235delC (p.L79Cfs∗3) mutation in *GJB2* is quite common and well characterized in East Asians, the c.481C>T (p.R161C) mutation in *SOX10* was far less frequent and its clinical manifestations were not consistent in different reports [32, 41].

In this study, the clinical manifestations of the family members carrying the c.481C>T (p.R161C) mutation in *SOX10* are distinct along three different generations: typical WS2 phenotype (hearing loss and hair hypopigmentation) in the paternal grandmother, hair hypopigmentation only in the father, and hearing loss only in the proband. The c.481C>T (p.R161C) mutation affected the HMG domain of SOX10, which is the sequence-specific DNA-binding domain, and was predicted to be damaging by in silico assessment.

The same *SOX10* c.481C>T (p.R161C) mutation has previously been reported in a Caucasian patient with Kallmann syndrome, in whom the presence or absence of hearing loss and pigmentation defect was not described (Table 1) [32]. Kallmann syndrome is a developmental disease that combines congenital hypogonadotropic hypogonadism with anosmia [42]. Our patients manifested hearing loss and hair hypopigmentation, but no anosmia or delayed puberty. This phenotypic difference suggests that other factors, such as modifier gene or epigenetic events, might contribute to the expression of the KS phenotypes. To date, the *SOX10* c.481C>T (p.R161C) mutation has been identified in six patients from three families (Table 1) [32, 41]. Hearing loss was observed in four of the six patients and seems to be a consistent feature in mutation carriers, while hypogonadism and anosmia symptoms were described only in one patient.

**Figure 3:**
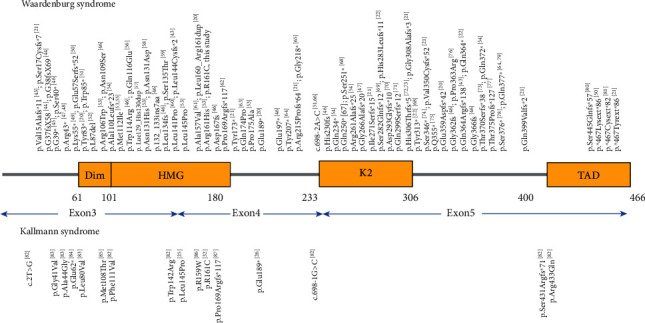
Schematic representation of the SOX10 domains and overview of *SOX10* mutations and their associated phenotypes.

Most of *SOX10* mutations are private and were identified in sporadic cases, making it difficult to correlate the genotypes with the distinct disease phenotypes. Herein, we identified a KS-associated *SOX10* mutation in a family with WS2, indicating that the same *SOX10* mutation can underlie both WS and KS. Among over 80 published *SOX10* mutations, three (p.Leu145Pro, p.Pro169Argfs∗117 and p.Glu189∗) were also found to lead to different phenotypes (Figure 3) [43–87]. Further investigation is needed to clarify the underlying mechanisms of incomplete penetrance and high phenotypic variability caused by *SOX10* mutations.

Our study also demonstrates that targeted NGS is a powerful strategy to discover causative genes in rare, heterogeneous disorders such as hearing loss. WS caused by *SOX10* mutations can resemble nonsyndromic hearing loss in young children who do not present with pigmentary abnormality. Targeted NGS has the potential to identify such mutations which would improve the management of hearing loss by genetic counseling for the children and risk assessment of the relatives.

## Figures and Tables

**Table 1:** Summary of clinical findings in patients with *SOX10* c.481C>T (p.R161C) mutation.

Patient	IV-1	III-1	II-1	Marcos et al.[32]	Bademci et al.[41]
Age	7 y	34 y	60 y	n.d.	9 y; 11 y
Gender	F	M	F	n.d.	F; M
Hearing loss/inner ear imaging					
Hearing loss	Profound	—	Profound	n.d.	Profound
Abnormal semicircular canal	Post. SCC dilatation	NA	NA	n.d.	n.d.
Pigmentation defects					
Pigmentary disturbances of iris	—	—	—	n.d.	n.d.
Iris heterochromia	—	—	—	n.d.	n.d.
Skin depigmentation	—	—	—	n.d.	n.d.
White forelock	—	+	+	n.d.	n.d.
Premature graying	—	+	+	n.d.	n.d.
Eye anomalies					
Telecanthus	—	—	—	n.d.	n.d.
Retinal pigmentation defect	—	—	—	n.d.	n.d.
Gastrointestine					
Constipation	—	—	—	n.d.	n.d.
Hirschsprung disease	—	—	—	n.d.	n.d.
Hypogonadotropic hypogonadism	—	—	—	+	n.d.
Delayed puberty	NA	—	—	+	n.d.
Anosmia or severe hyposmia	—	—	—	+	n.d.
Genetic					
*SOX10* mutation	c.481C>T	c.481C>T	c.481C>T	c.481C>T	c.481C>T

n.d.: not described; NA: not applicable; y: year.

## Data Availability

The data supporting the findings of this study are available within the article and the supplementary files.

## Supplementary Materials

Supplementary Materials Supplementary Table S1: a list of 415 deafness-related genes included in the targeted gene panel. Supplementary Table S2: candidate variants identified by targeted NGS in the proband VI-1 and her parents III-1 and III-2. Supplementary Table S3: primers used in this study.
